# The Fermentation Analogy: A Point of View for Understanding the Intriguing Role of Proline Accumulation in Stressed Plants

**DOI:** 10.3389/fpls.2016.01339

**Published:** 2016-08-31

**Authors:** Santiago Signorelli

**Affiliations:** ^1^School of Plant Biology and the UWA Institute of Agriculture, University of Western AustraliaCrawley, WA, Australia; ^2^Laboratorio de Bioquímica, Departamento de Biología Vegetal, Facultad de Agronomía, Universidad de la RepúblicaMontevideo, Uruguay

**Keywords:** photosynthesis, photorespiration, malate valve, proline metabolism, redox, abiotic stress, high light stress, reactive oxygen species

The accumulation of proline under environmental stress is a conserved response of plants. Five decades have passed since the first report of proline accumulation in plants (Barnnet and Naylor, 1966). Many hypotheses have been put forward regarding assignment of a function to proline. These proposed roles include antioxidant capacity, osmoprotection, signaling, developmental function, and contributing in redox and cellular homeostasis (Smirnoff and Cumbes, 1989; Mattioli et al., 2008; Szabados and Savouré, 2010; Kishor et al., 2015). Among these, the osmotic adjustment, osmoprotectant, and antioxidant role have probably been the earliest and most widely accepted functions of proline. It is known that compatible osmolytes contribute in the retention of water, but also some of them force proteins to adopt a compactly folded structure, preventing the unfolding and reducing the exposed surface of the protein to damaging compounds (Attri et al., 2010). In case of proline this was demonstrated both *in vitro* and in single cell organisms. For example, it was observed that proline overproducing *E. coli* mutants had greater osmotolerance (Csonka et al., 1988) and also proline can inhibit protein aggregation *in vivo* (Ignatova and Gierasch, 2006). However, neither osmotic adjustment nor osmoprotection has been clearly confirmed in plants (Maggio et al., 2002; Kavi Kishor and Sreenivasulu, 2014). This is probably more complex in plants because plants have different osmoregulatory mechanisms. Controversy is present regarding its proposed antioxidant role because recent evidence demonstrates that proline cannot scavenge singlet oxygen, superoxide, nitric oxide, peroxynitrite nor nitrogen dioxide (Signorelli et al., 2013, 2016). In view of the lack of activity in previously suggested roles it has recently been proposed that proline acts exclusively as a hydroxyl radical scavenger (Signorelli et al., 2014, 2015), but more evidence for the physiological importance of this role is needed.

Recently, the availability of proline biosynthesis Arabidopsis knock-out (KO) mutant plants has allowed researchers to provide direct evidence regarding the role of proline in plants. It was observed that one of these mutants (*p5cs2* mutant) were embryo lethal (Szekely et al., 2008) and that proline is required for flower transition and for pollen development and transmission (Mattioli et al., 2009, 2012; Funck et al., 2012). Additionally, it has been shown that proline content in roots correlates with root development (Sharma et al., 2011; Biancucci et al., 2015). This evidence clearly demonstrates the involvement of proline in development. However, the purpose of the accumulation of large amounts of proline in leaves under stressful conditions remains unresolved.

In a different approach Hare and Cress (1997) hypothesized that the key for proline accumulation could reside in the redox changes produced by the activation of proline metabolism. In particular, these authors suggested that proline catabolism might provide electrons to the mitochondrial electron transport chain while proline anabolism may contribute to regenerate cytoplasmic NADP^+^ useful for the pentose pathway. In this direction, De Ronde et al. (2004) showed that transgenic plants over-expressing P5CS have greater NADP^+^ contents. Later the hypothesis was extended suggesting that the greater regeneration of NADP^+^ would also be useful as the final electron acceptor of the photosynthetic electron transport chain (Szabados and Savouré, 2010). Accordingly, it was observed that Arabidopsis mutants, unable to accumulate proline, had a greater NADPH.H^+^/NADP^+^ ratio under condition of low water potential (Sharma et al., 2011). Moreover, these authors showed that proline catabolism in roots correlated with respiration rates in this organ (Sharma et al., 2011). This observation effectively demonstrated that proline metabolism has implications for important metabolic processes. Unfortunately, to the best of my knowledge there are no reports that relate the proline anabolism with photosynthetic activity cf. those for proline catabolism and respiration (Sharma et al., 2011). In my opinion, this should be evaluated because it could explain why proline is accumulated in such high levels and why it is a conserved response. In next paragraphs I will explain why I support this idea and suggest what could be evaluated to confirm it.

I consider that proline accumulation could be a consequence of the benefits produced by the activation of its metabolism more so than the molecule itself. In a similar way, during fermentation, prokaryotic and eukaryotic cells accumulate compounds (e.g., lactate, ethanol) to provide NAD^+^ necessary to continue with the glycolysis. Have these accumulated compounds a relevant role in the cell? I surmise that the answer is no.

In order to develop this idea, Figure 1 proposes an analogy between what happens in animals with lactic fermentation and proline accumulation in plants. Under anaerobic conditions animals cells (tumor or skeleton muscles cells) run out of NAD^+^ and glycolysis is stopped until it can be regenerated, because glycolysis requires it as much as glucose. Therefore, pyruvate is transformed into lactate to regenerate NAD^+^ and this process is called lactic fermentation (Nelson and Cox, 2016). The longer the demand of energy by glycolysis is, the greater is the accumulation of lactate. When conditions became optimum the accumulated lactate, is transported by blood to the liver to regenerate glucose and then returned to muscles (Cori cycle, Figure 1A). In a similar point of view, the high amount of proline accumulated in leaves could be a consequence of regenerating NADP^+^, necessary for the photosynthesis; an important biochemical process in plant, such as glycolysis is for non-photosynthetic organisms. Many stressful conditions produce low availability of NADP^+^ increasing the likelihood of photosynthetic electron leakage and reactive oxygen species (ROS) generation. Most common examples are: (i) high light intensity, which promotes high electron flux through the photosystem and therefore the NADP^+^ restrictions; and (ii) saline, osmotic and drought stress, which stimulate stomata closure, reducing the Calvin cycle activity and hence a low use of NADPH.H^+^. The hypothesis supported here implicates that in such conditions glutamate is reduced to proline in order to re-oxidize NADP^+^ and avoid interruption of the non-cyclic electron flow in the light phase of photosynthesis (Figure 1B). It would also prevent ROS production through photosynthetic electron leakage by reducing the time that electrons are stuck at the photosynthetic electron chain. Proline would then be oxidized to glutamate in mitochondria to generate NADH.H^+^, FADH_2_ or donating its electrons directly to the respiratory chain to produce ATP (Figure 1B). Part of this oxidation would take place in the photosynthetic tissue, but also it is known that the excess of amino acids produced in leaves is exported via the phloem to others organs (Fischer, 1998). So, proline would be also redistributed to different organs, such as roots, to be oxidized. Lastly, considering that amino acids are the main long distance transport form of organic nitrogen (Wipf et al., 2002; Näsholm et al., 2009) and glutamate and glutamine are two of the most common amino acids found in the vascular tissues (Fischer, 1998), it would be expected that glutamate returns via xylem to the leaves either as glutamate, or glutamine if the uptake of nitrogen by roots is good (Figure 1B).

**Figure 1:**
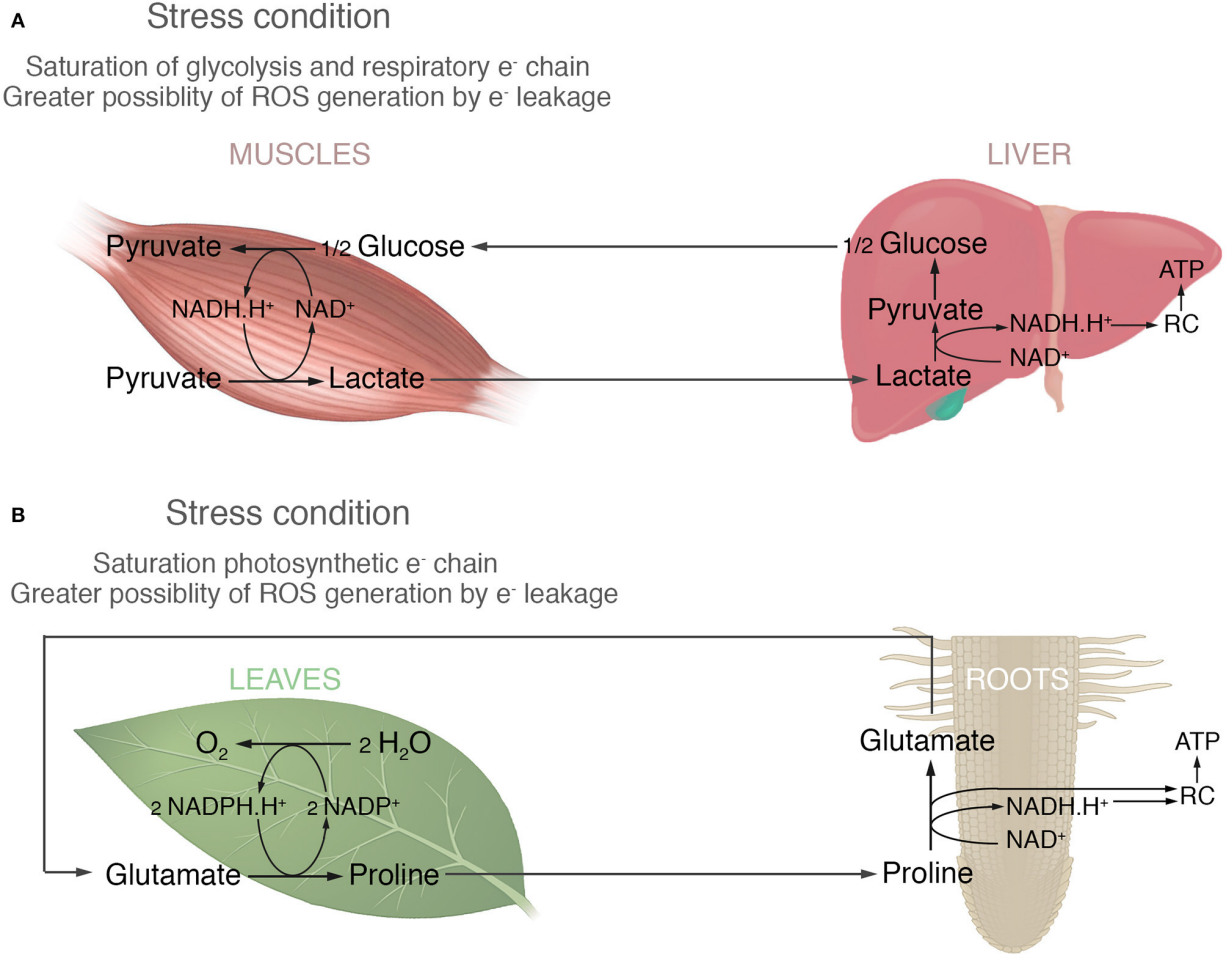
**The analogy between animal fermentation and proline accumulation in plants**. **(A)** In animals the high activity of muscles requires elevated rates of glycolysis. In this situation, the generation of NADH.H^+^ by glycolysis overcomes the respiration chain's capacity to consume it. Therefore, the pyruvate produced by glycolysis is reduced to lactate in order to re-oxidize the excess of NADH.H^+^ and avoid the interruption of glycolysis due to a lack of NAD^+^. The accumulated lactate is transported to the liver and reconverted to pyruvate regenerating the NADH.H^+^ which, in turn, can be used to produce ATP by the respiratory chain (RC). This ATP is then used to regenerate glucose. **(B)** In plants photosynthetic activity requires NADP^+^ as the final electron acceptor. Different stresses might result in a lower availability of NADP^+^ leading to photosynthetic electron leakage becoming more likely. In this case, glutamate is reduced to proline in order to re-oxidize two NADPH.H^+^ and avoid the interruption of photosynthesis due to a lack of NADP^+^. It also by reducing the time that electrons are stuck at the photosystem, prevents ROS generation by photosynthetic electron leakage. The accumulated proline could be then oxidized to glutamate in the mitochondria of different plant organs providing reducing power for the production of ATP in the respiratory chain (RC).

Since many stresses affect CO_2_ assimilation but not the electron transport capacity at the photosystem, there are different mechanisms to consume the electrons derived from water oxidation (Lawlor and Cornic, 2002). Among them, the photorespiration, Mehler reaction and respiratory chain use oxygen as electron acceptor and are probably the most relevant. In case of the respiratory chain, the electron should be transferred to the mitochondria and, as described above, proline could be implicated in such process. Another mechanism is the nitrate reduction, but considering that nitrate and nitrite reductase activities are inhibited by stress (Krishna Rao and Gnanam, 1990), it would not be relevant in the dissipation of reducing power under stress.

Returning to the previous mentioned mechanism, some advantages of the proline accumulation are discussed below. In the Mehler reaction the photosystem I transfers the electrons to oxygen producing superoxide anion (O${}_{2}^{\cdot -}$). This ROS is able to damage biomolecules, so different antioxidant mechanism are needed to control its toxicity. The proline accumulation not only does not produce ROS as Mehler reaction does, but also it can both scavenge hydroxyl radical and act as osmoprotectant. In case of photorespiration, energy (ATP), NH_3_ and CO_2_ are wasted, making this process not very convenient for the plant. Opposite photorespiration, by accumulating proline the cells take advantage of the excess of reducing power and use it in the mitochondrial respiratory chain. It is worth mentioning that even under stress condition proline is degrade during night period (Sanada et al., 1995; Hayashi et al., 2000). In this assumption, proline accumulation would act in the same direction than the malate valve. In this process chloroplastic NADPH.H^+^ is consumed by the NADP-dependent malate dehydrogenase to convert oxaloacetate into malate and then malate is transported to the mitochondria to be converted back in oxaloacetate by the NAD-dependent malate dehydrogenase and producing NADH.H^+^ useful for the respiratory chain. If these process act in the same direction it would be expected that the lack of one mechanism enhanced the activity of the other. Interestingly, Arabidopsis plants lacking the NADP-malate dehydrogenase have similar content of proline under normal light condition (50 μmol^.^m^−2.^s^−1^) but enhanced proline accumulation under high light treatment (750 μmol^.^m^−2.^s^−1^) (Hebbelmann et al., 2012). This might indicate that proline is acting as a compensatory mechanism for the malate valve. Considering that proline transporters are upregulated under several stress conditions (Rentsch et al., 1996; Ueda et al., 2001), perhaps the malate valve plays a more relevant role at the intracellular whereas the proline plays a more relevant role distributing the reducing power between different organs (Figure 1B).

In this scenario, it would be feasible to establish whether the malate valve or the proline accumulation will provide more chloroplastic NADP^+^. The enzymes involved in these pathways have similar K_M_ values for NADPH.H^+^, 0.05–0.03 mM and 0.045 mM, P5CR and NADP-MDH respectively (Szoke et al., 1992; Lemaire et al., 1996; Giberti et al., 2014), but the P5CS K_M_ for NADPH.H^+^ is not known. So, the characterized enzymes involved in these processes have similar affinity for NADPH.H^+^. Regarding the affinity for their substrates, the P5CS has a K_M_ of 3.6 mM for glutamate (Zhang et al., 1995), the P5CR has a K_M_ of 0.15–0.10 mM for P5C (Szoke et al., 1992; Giberti et al., 2014) and the NADP-MDH has a K_M_ of 0.04 mM for oxaloacetate (Lemaire et al., 1996). Despite the K_M_ of NADP-MDH being lower for its substrate, to compare the likelihood of these process the concentration of the substrates should be consider. Taking into account that in stroma, the oxaloacetate and malate levels are below 0.2 and 0.4 mM respectively (Douce, 1985; Büssis and Heineke, 1998) and the glutamate and proline concentrations are about 47 and 120 mM respectively (Büssis and Heineke, 1998), it would be expected that proline biosynthesis is as important as the malate valve in the regeneration of chloroplastic NADP^+^. However, the comparison of proline to the malate valve in regenerating chloroplast NADP^+^ is something that needs further investigation, preferably using data from the same plant species.

Evidence supporting the potential role of proline accumulation in photosynthesis and the analogy with fermentation:

- Proline is biosynthesized in photosynthetic tissues primarily. Despite the demonstration that proline is accumulated also in other organs, it is suggested that the biosynthesis under osmotic stress is greater in leaves. For example, in Arabidopsis the expression of *P5CS1* gene is greater in leaves than flowers (Szekely et al., 2008). Also, the P5CS1 protein contents are low in root tips of Arabidopsis seedlings, but high in cotyledons and leaf primordia (Szekely et al., 2008). In concordance, in maize roots proline synthesis from glutamate remains constant at low water potential, however proline uptake increases, suggesting that proline transport play an important role in proline accumulation in roots (Verslues and Sharp, 1999).
- Proline accumulation is light dependent (Abrahám et al., 2003; Díaz et al., 2005), as the light phase of photosynthesis is. Proline accumulation is high in light and low in dark in plants subjected to saline stress and 12 h light/12 h dark cycle (Sanada et al., 1995). Moreover, the proline accumulation in leaves of barley treated with saline stress is enhanced upon 4 days of continuous light and suppressed after 4 days of continuous dark (Fedina et al., 2002). Concordantly to the proline contents, the protein and mRNA levels of P5CS and ProDH oscillate in the light/dark cycles with a reciprocal relationship (Hayashi et al., 2000). Moreover, under continuous dark P5CS is not expressed through the time course, but it is expressed under continues light and increasing over the time (Hayashi et al., 2000).
- The greatest accumulation of proline occurs into chloroplast (Büssis and Heineke, 1998). It could be explained because, the inducible enzyme responsible of proline accumulation, P5CS1, is suggested to be accumulated at the chloroplasts under stress condition (Szekely et al., 2008). Interestingly, in control conditions this enzyme is expressed in both cytoplasm and chloroplasts, but re-localized to chloroplast just under stress condition (Szekely et al., 2008).

Together (i), (ii), and (iii) suggest a spatial-temporal coincidence between photosynthesis and proline accumulation, as occurs with glycolysis and fermentation.

(iv) Recently, it was suggested that chloroplast and mitochondrial electron transport in the mutant deficient in proline accumulation, *p5cs1-4*, are altered (Shinde et al., 2016). Interestingly, the authors evaluated the RNA levels of *p5cs1-4* and they found that most of the highly up-regulated genes are chloroplast encoded genes. Moreover, most of these are genes involved in the light reactions of photosynthesis (Shinde et al., 2016). Additionally, there are few reports showing that transgenic plants with greater accumulation of proline have better photosynthetic capacity (Vendruscolo et al., 2007; Surender Reddy et al., 2015). The finding that proline over-accumulating plants have much greater maximum photochemical efficiency of PSII than wt plants under saline stress (Surender Reddy et al., 2015) together with recent findings of Shinde et al. (2016) clearly express that proline accumulation has implications in the light phase of photosynthesis.
(v) Under stress conditions the transport of proline along the plant is increased. In alfalfa the proline concentration in the phloem is increased by 60 folds upon water stress (Girousse et al., 1996). In Arabidopsis the genes coding for proline transporters, *ProT1* and *ProT2*, are expressed in all organs, with highest expression in roots, and *ProT2* is strongly induced in leaves under both water and salt stress (Rentsch et al., 1996). Moreover, in barley, the gene for the proline transporter *HvProT* is over expressed in root tips under salt stress (Ueda et al., 2001). In addition to water and saline stress, in *Chrysanthemum lavandulifolium*, some proline transporters are over-expressed upon cold and heat stress (Zhang et al., 2014).

Other evidence also supports a role for proline in controlling redox-status. Very recently it was observed that lipid metabolism mutants have high proline accumulation under stress, and the greater proline accumulation is caused mainly by the altered redox status of these mutants (Shinde et al., 2016). Finally, the linkage between proline accumulation and developmental process, could be also related with the control of redox-balance. It is known that developmental processes are tightly regulated by redox states (Foyer and Noctor, 2011). Moreover, the P5CR activity, involved in proline biosynthesis, is regulated by the redox status of the pyridine nucleotides (Giberti et al., 2014). However, the fact that P5CR be directly involved in supporting chloroplastic NADP^+^ could be questioned now, because despite evidence reporting P5CR in chloroplast (Kohl et al., 1988; Szoke et al., 1992), a more recent study has demonstrated that in Arabidopsis this enzyme is located exclusively in the cytoplasm (Funck et al., 2012).

Further studies will be necessary to probe, under stress conditions, whether proline accumulation has direct repercussions for photosynthetic activity. In order to do this mutants deficient in proline accumulation are clearly a valuable tool. However, because there are many process controlling the chloroplastic NADPH.H^+^/NADP^+^ ratio, it would be interesting to evaluate if mutants for these process accumulate more proline as a compensatory mechanism. For example, we now know that mutants for the malate valve accumulate more proline under high light intensity (Hebbelmann et al., 2012). But, do photorespiratory or cyclic electron flow mutants accumulate more proline? Or, do non-proline accumulation mutants more photorespiration? Additionally, it might be good to cross non-proline accumulating mutants with mutants for these other processes in order to visualize greater effects.

The photosynthetic activity and related parameters should be assessed in these mutants subjected to either osmotic deficit or excess of light. These conditions produce proline accumulation and also an imbalance between the carbon fixation and the electron flux at the photosystem. Additionally, more evidence about the P5CS and P5CR localization would be helpful to improve our understanding of proline anabolism and evaluate whether proline biosynthesis is implicated in the consuming of photosynthetic NADPH.H^+^.

Finally, it would be helpful to evaluate the transport of proline and glutamate. For example, it would be interesting evaluate the proline contents in roots and leaves of mutants for the transport of proline (e.g., some *AtProT* KO mutant), and the glutamate contents in leaves and roots of mutants for the transport of glutamate (e.g. the *AtAPP6* KO mutant) subjected to osmotic deficit. Considering the cycle of Figure 1B, proline transporter mutants are expected to have greater proline in leaves and lower in roots respect to wt. In case of glutamate transporter mutants, lower levels of glutamate in leaves and greater in roots are expected. However, the redundancy for amino acids transporters could be a problem to visualize differences and the use of multiple KO mutants might be required.

Summarizing, the accumulation of proline in green tissues of plants could be a mechanism to avoid photo-inhibition in a manner similar to that of accumulation of lactate promotes glycolysis to avoid muscular failure. Here I propose a range biological tools and stress conditions that could aid understanding of the role of proline accumulation in protecting photosynthesis under stress conditions.

## Author contributions

The author confirms being the sole contributor of this work and approved it for publication.

### Conflict of interest statement

The author declares that the research was conducted in the absence of any commercial or financial relationships that could be construed as a potential conflict of interest.
